# Structural basis for spermidine recognition and modulation of *Acinetobacter baumannii* multidrug efflux regulator AmvR

**DOI:** 10.1128/mbio.00081-25

**Published:** 2025-03-31

**Authors:** Na Wang, Xu Wang, Mengxiang Zhou, Qingsong Lu, Yaling Xu, Ying Wang, Haiyun Wang, Beibei Yang, Shibing He, Liuliu Xu, Jie Li, Honghua Ge, Jinming Ma

**Affiliations:** 1 Institute of Health Sciences and Technology, Institutes of Physical and Information Technology, Anhui University 606378 , Hefei, Anhui, China; Columbia University, New York, New York, USA

**Keywords:** Multidrug efflux regulator, spermidine, substrate recognition

## Abstract

*Acinetobacter baumannii* is a gram-negative, opportunistic pathogen frequently associated with hospital-acquired infections. Due to its resistance to multiple antibiotics, it is emerging as a major nosocomial pathogen, causing a wide range of severe infections such as pneumonia, meningitis, and bloodstream infections. In many cases, the intrinsic activities of efflux pumps contribute to the development of drug resistance. The polyamine-binding protein AmvR regulates the multidrug efflux pump AmvA, which is pivotal for transporting polyamines, an abundant and prevalent class of amino acid-derived metabolites. Here, we report the crystal structure of the AmvR protein bound to its physiological substrate, spermidine, thereby offering structural and functional insights into AmvR. By employing electrophoretic mobility shift assays and DNase I footprinting, we identified the recognition sites of the intragenic regions of *amvR* and *amvA* by AmvR. Moreover, a fluorescence reporter assay revealed that AmvR repressed the expressions of AmvA and AmvR. In addition, isothermal titration calorimetry indicated that spermidine may be a natural ligand of AmvR. Collectively, these experiments provided a better understanding of substrate recognition for the discovery of potential inhibitors. Furthermore, our results revealed that substrate binding triggers a localized conformational change in the AmvR protein, as supported by size-exclusion chromatography and static light scattering, suggesting a distinctive regulatory mechanism within the TetR family transcription factors.

**IMPORTANCE:**

Multidrug efflux pumps are key contributors to clinically significant drug resistance in various gram-negative pathogens responsible for hospital-acquired infections. These pathogens often possess multiple genes that encode potential multidrug efflux pumps. Identifying the specific regulatory proteins that control the expression of these pumps, along with elucidating the regulatory mechanisms triggered by effectors, presents a complex challenge. In this study, we have resolved the crystal structures of AmvR in both its unbound and spermidine-bound states. To the best of our knowledge, this represents the first validated structural model of a polyamine-bound transcriptional regulator. Through detailed structural analysis and functional assays, we have pinpointed the critical residues in AmvR responsible for substrate recognition, providing a foundation for the development of future inhibitors.

## INTRODUCTION

*Acinetobacter baumannii* is a prominent nosocomial pathogen that poses a substantial threat to human health (1). As a principal opportunistic pathogen, it primarily colonizes intensive care units (ICUs) and is often involved in coinfections and secondary infections in patients (2). The notorious status of *A. baumannii* as an “ESKAPE” pathogen (*Enterococcus faecium*, *Staphylococcus aureus*, *Klebsiella pneumoniae*, *Acinetobacter baumannii*, *Pseudomonas aeruginosa*, and *Enterobacter* spp.) results from its remarkable genetic adaptability and multidrug efflux mechanisms, which lead to the emergence and continuous increase in antibiotic resistance (3–6). Furthermore, *A. baumannii* is proficient in forming biofilms, protective layers of extracellular matrices secreted by bacterial colonies, that facilitate its adherence to both biotic and abiotic surfaces within hospital settings, thereby complicating eradication efforts and amplifying transmission risks (7–9).

Polyamines, a class of small, positively charged molecules derived from amino acids, are ubiquitous in all living organisms (10, 11). These molecules, including putrescine, cadaverine, spermidine, and spermine, play essential roles in multiple cellular functions across all the domains of life (10, 12). Their contributions are critical to nucleic acid stabilization, chromatin remodeling, and the regulation of gene expression, as well as cell growth, differentiation, and stress response mechanisms (12, 13). In bacteria, polyamines are particularly important, aiding in nitrogen metabolism (14), cell proliferation, virulence, and biofilm development (10, 15, 16). In *Escherichia coli* and possibly other bacteria, polyamines are required for base modification in tRNA anticodons (17).

Cellular polyamine levels are rigorously controlled by intricate regulatory processes that maintain optimal concentrations and mitigate potential toxicity (18). Transcription factors, in conjunction with environmental cues, orchestrate the regulation of key enzymes involved in polyamine biosynthesis at the transcriptional level, supported by allosteric feedback mechanisms, to preserve polyamine homeostasis (19, 20). Furthermore, bacterial cells fine-tune the uptake of polyamines through dedicated transport systems that enable the absorption of exogenous polyamines (21). Dysregulation of polyamine metabolism in bacteria can adversely affect various cellular processes, including growth, stress response, and virulence, highlighting the importance of understanding the mechanisms underlying polyamine regulation when devising antimicrobial strategies (22).

In *A. baumannii*, several efflux systems assist the transport of polyamines. AmvA belongs to the major facilitator superfamily (MFS) of transporters (23). In 2022, Short et al. demonstrated that AmvA is particularly instrumental in expelling long-chain polyamines such as spermidine and spermine from cells (24). This efflux activity enables *A. baumannii* to withstand common hospital disinfectants, including chlorhexidine and benzalkonium chloride (1). AmvA is also implicated in biofilm development and virulence by facilitating the secretion of polyamines or other virulence factors (5, 7). AmvA abundance *in vivo* is transcriptionally regulated by AmvR, a TetR/AcrR-like regulator that senses cellular polyamine levels and enables the dynamic modulation of *amvA* expression in response to fluctuating growth conditions (24). However, the precise structural mechanisms underlying the regulatory relationship between AmvA and AmvR remain elusive, impeding our comprehensive understanding of the pivotal functional role of AmvR in *A. baumannii*’s response to polyamines.

In this study, we report the crystal structures of both ligand-free and spermidine (SPD)-bound AmvR. In its ligand-free state, the DNA-binding domains of the AmvR dimer adopt a dimeric arrangement, maintaining their association, but exhibiting a long distance between the two DNA recognition helices, similar to the arrangement observed in the ligand-bound state. Upon binding to its inducer spermidine, AmvR undergoes conformational changes, rendering it unable to bind to the promoter region of AmvA, thus unblocking transcription. We also analyzed the polyamine binding site of AmvR to identify the essential residues involved in substrate recognition. Furthermore, DNase I footprinting and electrophoretic mobility shift assays (EMSAs) were employed to map the recognition sites of AmvR within the intragenic regions of *amvR* and *amvA*. Our work represents an initial step toward understanding the structural basis underlying the functionality of the regulatory AmvR protein that governs both drug resistance and virulence in *A. baumannii*.

## RESULTS

### AmvR is a TetR family transcription regulator

**Fig 1:**
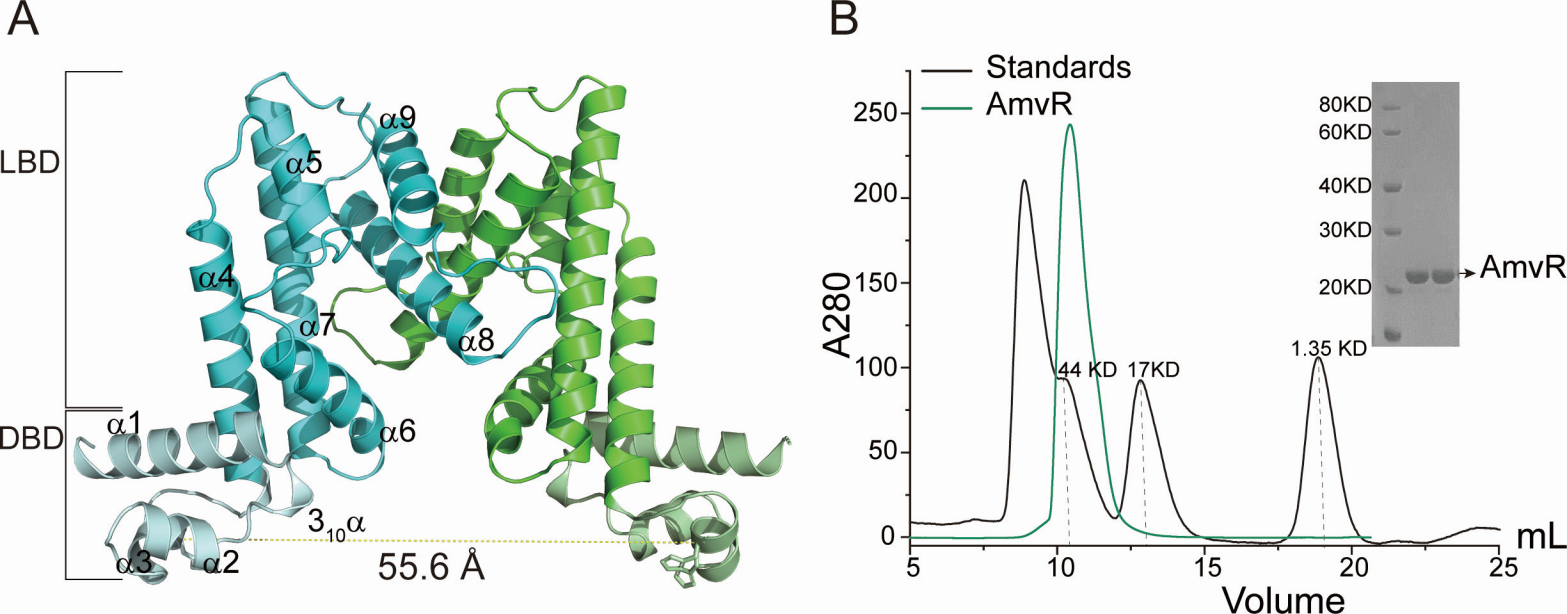
Properties of AmvR protein from *A. baumannii*. (A) The structure of *apo* AmvR dimer. The protomers are depicted in cyan and green, respectively. The helices α3 and α4 constituting the HTH DNA binding domain are marked. (B) Size-exclusion chromatography analysis of the *apo* AmvR was a dimer in solution. The standard sample (thyroglobulin 670 kDa; γ-globulin 158 kDa; ovalbumin 44 kDa; myoglobin 17 kDa; vitamin B12 1.35 kDa) was separated on the same column shown in black.

AmvR has been reported to exhibit sensitivity to increased levels of exogenous polyamines, such as putrescine, cadaverine, spermidine, and spermine and regulate the expression of the corresponding efflux protein AmvA (24). To understand AmvR’s response mechanism to polyamine, we first determined the crystal structure of an AmvR protein derived from *A. baumannii* at a resolution of 1.88 Å (as shown in Table 1). A single AmvR polypeptide chain is situated within the asymmetric unit of the crystal and forms a homodimer structure with its corresponding partner in another crystallographic lattice, which is consistent with the results of size-exclusion chromatography (Fig. 1A and B). The overall fold change observed in AmvR is characteristic of the TetR/AcrR family of transcription factors and consists of an all-helical regulatory domain (α1–α9) (Fig. S1). The AmvR protein can be further divided into two subdomains in each protomer: the N-terminal DNA-binding domain (DBD) and the C-terminal ligand-binding domain (LBD). Dimerization occurs primarily through hydrophobic interactions between helices α8 and α9 within the LBD (Fig. S2). The G162R substitution at the dimer interface, despite causing some protein instability, drove AmvR to adopt a monomeric state. This conformational shift was reflected by changes in retention time (Fig. 4C), which corresponded to a decrease in radius, as demonstrated by static light scattering (Fig. S3B). However, no significant change was observed in the secondary structure (Fig. S3C), indicating that the folding state of the protein remained unaffected.

**TABLE 1:** Data collection and refinement statistics

Parameter	*apo*-AmvR	AmvR-SPD
Data collection		
Wavelength (Å)	0.97918	0.97918
Space group	P 2_1_ 2_1_ 2	P 2_1_ 2_1_ 2
*a, b, c* (Å)	64.15, 69.60, 50.77	60.01, 65.51, 101.03
*α, β, γ* (°)	90.00, 90.00, 90.00	90.00, 90.00, 90.00
Resolution (Å)	50.77–1.87 (1.97–1.87)^*a*^	50.00–2.50 (2.56–2.50)
Total No. of reflections	171,627 (12,657)	163,932 (8,747)
No. of unique reflections	18,445 (1,921)	14,340 (1,022)
Completeness (%)	94.6 (70.3)	100.0 (99.6)
Average I/σ	16.1 (2.7)	20.6 (1.7)
CC_1/2_	0.997 (0.843)	0.995 (0.572)
*R*merge	0.076 (0.596)	0.173 (1.385)
*R*meas	0.080 (0.645)	0.181 (1.475)
*R*pim	0.025 (0.237)	0.054 (0.496)
Redundancy	9.3 (6.6)	20.6 (1.7)
Wilson B-factor (Å)	34.1	53.2
Refinement		
Resolution (Å)	50.77–1.87	50.00–2.50
No. of reflections	18,409 (1,252)	14,295 (1,377)
*R*_work_/*R*_free_	0.200/0.229	0.234/0.268
No. of non-hydrogen atoms	1,591	3,001
Protein	1,495	2,981
Ligand	0	20
Water	96	0
Average B-factors (Å^2^)	44.29	79.14
Protein	44.01	79.21
Ligand	-	68.92
Water	48.68	-
R.m.s.d. from ideality		
Bond lengths (A°)	0.005	0.004
Bond angles (°)	1.05	0.87
Ramachandran plots		
Favored (%)	98.36	97.83
Allowed (%)	1.64	2.17
Outliers (%)	0.00	0.00
PDB Code	8WP6	9IPT

^*a*^ Values in parentheses are for the highest-resolution shell. One crystal was used for each data set.

The second and third helices of the DNA-binding domain exhibit a classical helix-turn-helix (HTH) motif, with the recognition helices (α2) playing a pivotal role in sequence-specific DNA binding and being separated by 55.6 Å in dimeric form (measured as the distance between main-chain carbon atoms of His46) (Fig. 1A). Although the majority of ligand-free and DNA-bound TetR/AcrR-like regulators exhibit distances ranging from 35 to 41 Å, suiting the space between the half-sites in the operator (25–27), *apo* AmvR displays longer distances similar to those usually observed in the ligand-bound state. Therefore, we speculated that AmvR may have different states in solution, including *apo*-state and ligand-bound state, but we only captured one state in the crystal (28).

### AmvR regulates the expression of AmvA

**Fig 2:**
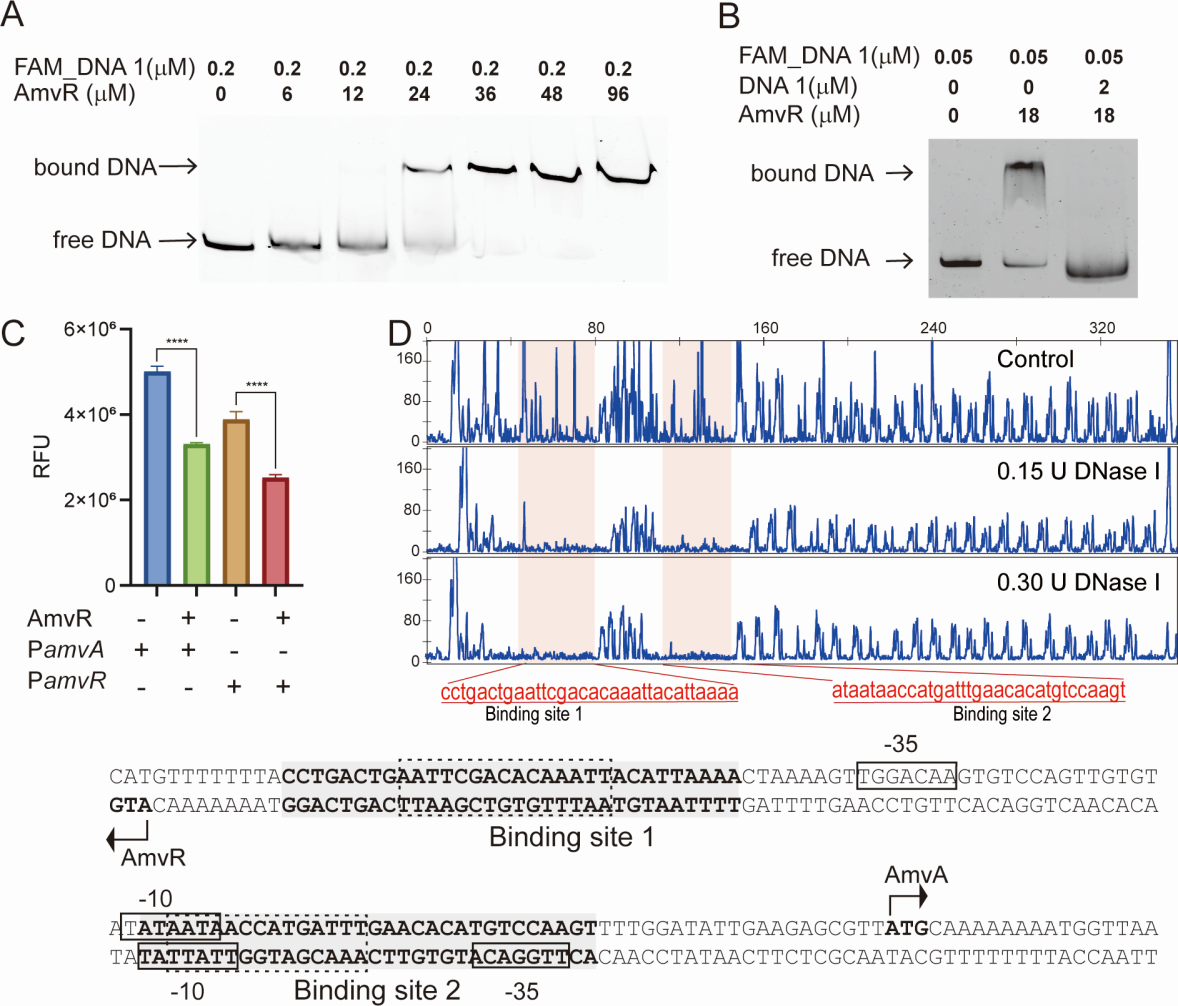
Identification of AmvR binding sites in the intergenic region of *amvR–amvA*. (A) The EMSA result of AmvR with intergenic region. (B) Unlabeled DNA was added to inhibit the binding of AmvR and the intergenic region of *amvR–amvA*, and nonspecific DNA was added as a negative control. (C) The fluorescence reporting assay of AmvR with the promoter of *amvR* and *amvA* illustrates that AmvR can repress the expression of *amvR* and *amvA*. (D) DNase I footprinting assay of AmvR with the *amvR–amvA* intergenic region. Gray-shaded boxes indicate DNA regions protected by AmvR. Boxes indicate putative −10 and −35 regions. Dark dash indicates the palindromic sequences.

Bacterial regulators of multidrug efflux play a pivotal role in modulating the expression of multidrug efflux pumps by binding to specific sequences within their promoter regions, and AmvR has been reported to repress AmvA (24). To verify that AmvR could bind to the promoter region, we performed EMSAs of AmvR with the FAM-labeled intergenic region of *amvA* and *amvR,* a 353 bp DNA denoted as FAM_DNA 1 (Table S1). We found that AmvR bound to FAM_DNA 1, leading to a clear shift in the bands (Fig. 2A). The shift bands were abolished by adding unlabeled DNA 1 (Table S1), indicating that the interaction between AmvR and the promoter region of *amvA* and *amvR* was specific (Fig. 2B).

We then conducted a DNase I footprinting assay with 353 bp DNA 1 to determine the binding site of AmvR. We found two regions in DNA 1 that were protected by increasing concentrations of DNase I (Fig. 2D). Analysis of the protected regions revealed two similar palindromic sequences, consistent with previous findings that the TetR family regulators usually form homodimers and bind to palindromic sequences (Fig. 2D) (29). The binding site 1 was located upstream of the −35 region for *amvA*, whereas the binding site 2 overlapped with the −10 regions of both *amvA* and *amvR* (Fig. 2D). Thus, we speculate that AmvR represses the transcription of the *amvR* operon and *amvA* by blocking the attachment of the RNA polymerase to their promoters, thereby preventing transcription initiation and extension.

To determine whether AmvR directly regulates the expression of *amvA* and *amvR*, we conducted a fluorescence reporter assay. We constructed a reporter plasmid with the promoter region of *amvR* or *amvA*, followed by the *egfp* gene combined with or without *amvR. E. coli* strains containing P*amvR*-EGFP or P*amvA*-EGFP displayed higher fluorescence than those containing AmvR-P*amvR*-EGFP or AmvR-P*amvA*-EGFP (Fig. 2C). The expression levels of EGFP confirmed that AmvR functions as a transcription factor, suppressing the expression of both *amvA* and *amvR* by interacting with their promoter regions.

### Spermidine is a natural ligand of AmvR

**Fig 3:**
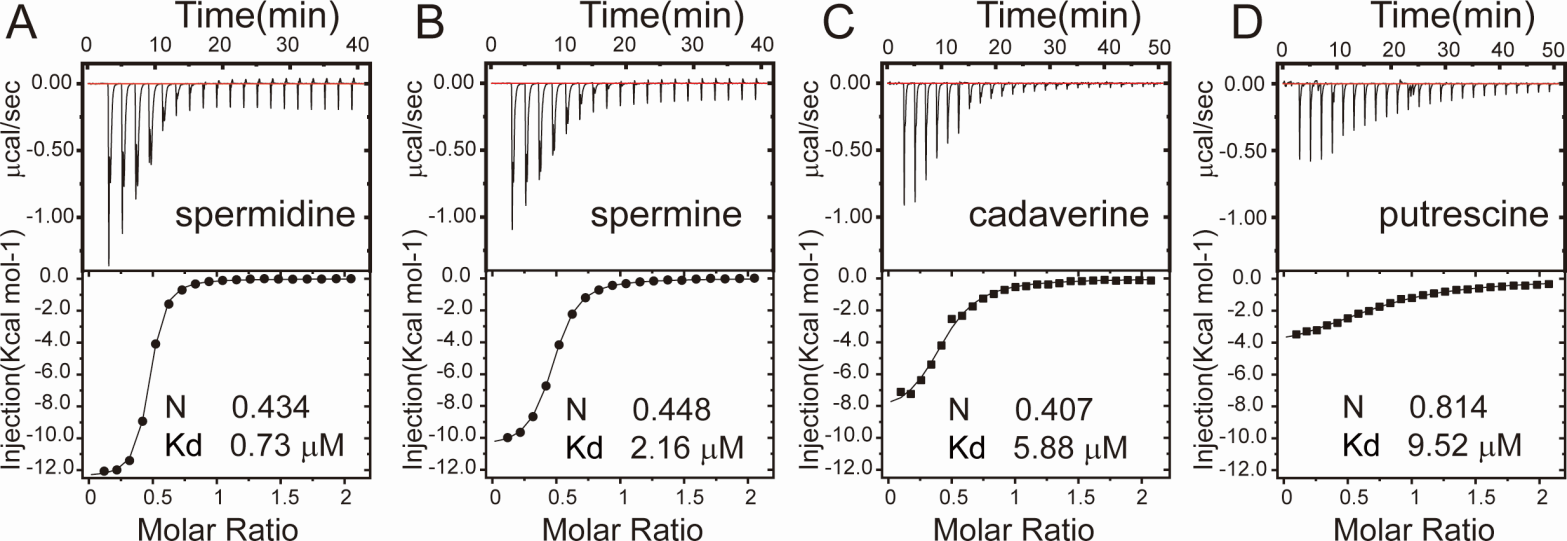
Isothermal titration calorimetry experiment shows the preference of AmvR with polyamine. (A–D) The ITC of spermidine (A), spermine (B), putrescine (C), and cadaverine (D) with *apo* AmvR. The stoichiometry (N) and the equilibrium dissociation (Kd) constant are denoted.

Because the disinfectant resistance protein AmvA functions as a polyamine efflux pump, it was hypothesized that long-chain polyamines induce the AmvR regulon (28). To validate this hypothesis and determine the binding affinities of the polyamines for AmvR, we employed isothermal titration calorimetry (ITC) (Fig. 3). The equilibrium dissociation constant (Kd) indicated that spermidine had the highest affinity for AmvR (0.73 µM) (Fig. 3A). This value was significantly higher than spermine, cadaverine, and putrescine for AmvR, which exhibited Kd values of 2.16 µM, 5.88 µM, and 9.52 µM (Fig. 3B through D). This observation is consistent with the previously reported findings that spermidine and spermine are more effective at elevating the thermal stability of AmvR (24). The binding constants determined using nanoDSF were compared with those obtained from ITC experiments (24). Therefore, we predicted that spermidine is a natural signaling molecule that can induce the expression of the polyamine efflux pump AmvA.

### Co-crystal structures of AmvR with spermidine

**Fig 4:**
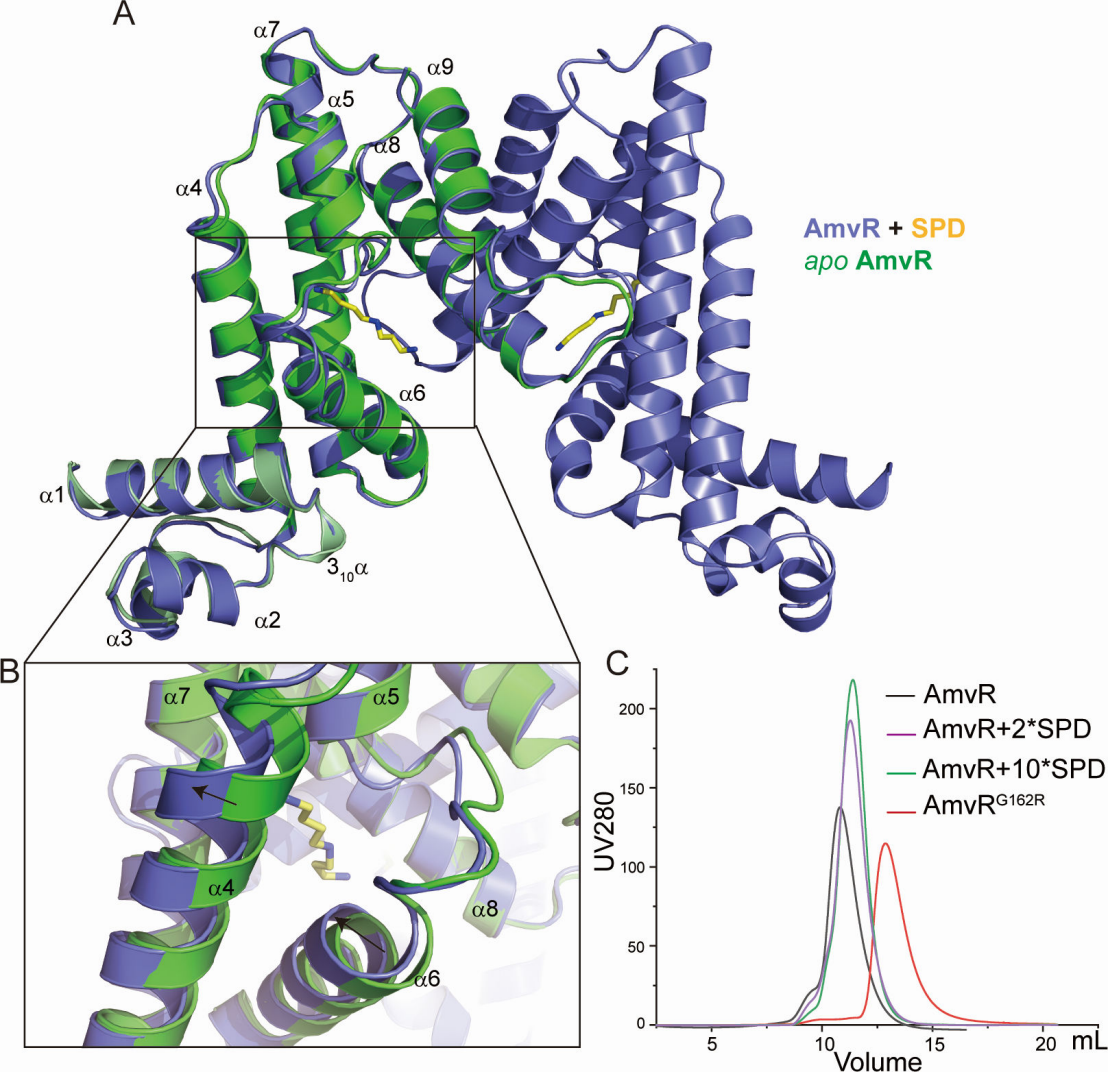
Comparison of the *apo* and SPD-bound AmvR structures. (A) Superposition SPD-bound AmvR dimer (colored in light blue) and free AmvR (colored in green). Spermidine is shown in yellow sticks. (B) The outward movement of α5 and inward movement of α7 is indicated by an arrow. (C) Size-exclusion chromatography illustrates that AmvR bound to spermidine has a longer retention time, whereas the substitution of G162 with arginine causes AmvR to adopt a monomeric form.

Based on the structural characteristics of *apo* AmvR, a TetR/AcrR family regulator, substrate binding is mediated by the LBD of this protein. We purified the AmvR with spermidine in solution and found that spermidine caused AmvR to display a longer retention time (Fig. 4C). The discrepancy in retention time became more pronounced as spermidine concentration increased but differed from that of AmvR^G162R^ (Fig. 4C). Static light-scattering experiments revealed a reduction in radius after incubation with spermidine (Fig. S3A). The results of size-exclusion chromatography (SEC) combined with static light scattering illustrated that the binding of spermidine causes AmvR to undergo a substantial conformational transformation (Fig. 4C).

We successfully determined the crystal structure of AmvR complexed with spermidine to precisely investigate the substrate-binding pocket (Fig. 4A and B). Two AmvR polypeptide chains were situated within the asymmetric unit of the crystal and formed a homodimer. However, the overall structure of this TetR family regulator remains largely unchanged between the *apo* and SPD-bound states, unlike other regulators that exhibit substantial structural differences. The structures can be superposed with the root mean square deviation values of 0.697 Å on 168 Cα atoms. Notable alterations were observed only in α4 and α6 of the LBD subdomain (Fig. 4B). The α4 undergoes a movement of approximately 2.8 Å to create sufficient space for ligand binding, and the α6 also moves to enhance compactness (Fig. 4B). The high-resolution structure indicated that the stoichiometry of spermidine binding to AmvR was a 1:1 ratio (one molecule of spermidine present in each protomer of AmvR). TetR family regulators typically bind ligands either in the pocket of each protomer or within a larger tunnel formed by the pocket of each protomer (28, 30). Here, the binding pocket of AmvR, located within each promoter, forms a narrow tunnel rather than a large one, which aligns with the elongated structure of polyamine substrates (Fig. S4A).

### Binding pocket of AmvR

**Fig 5:**
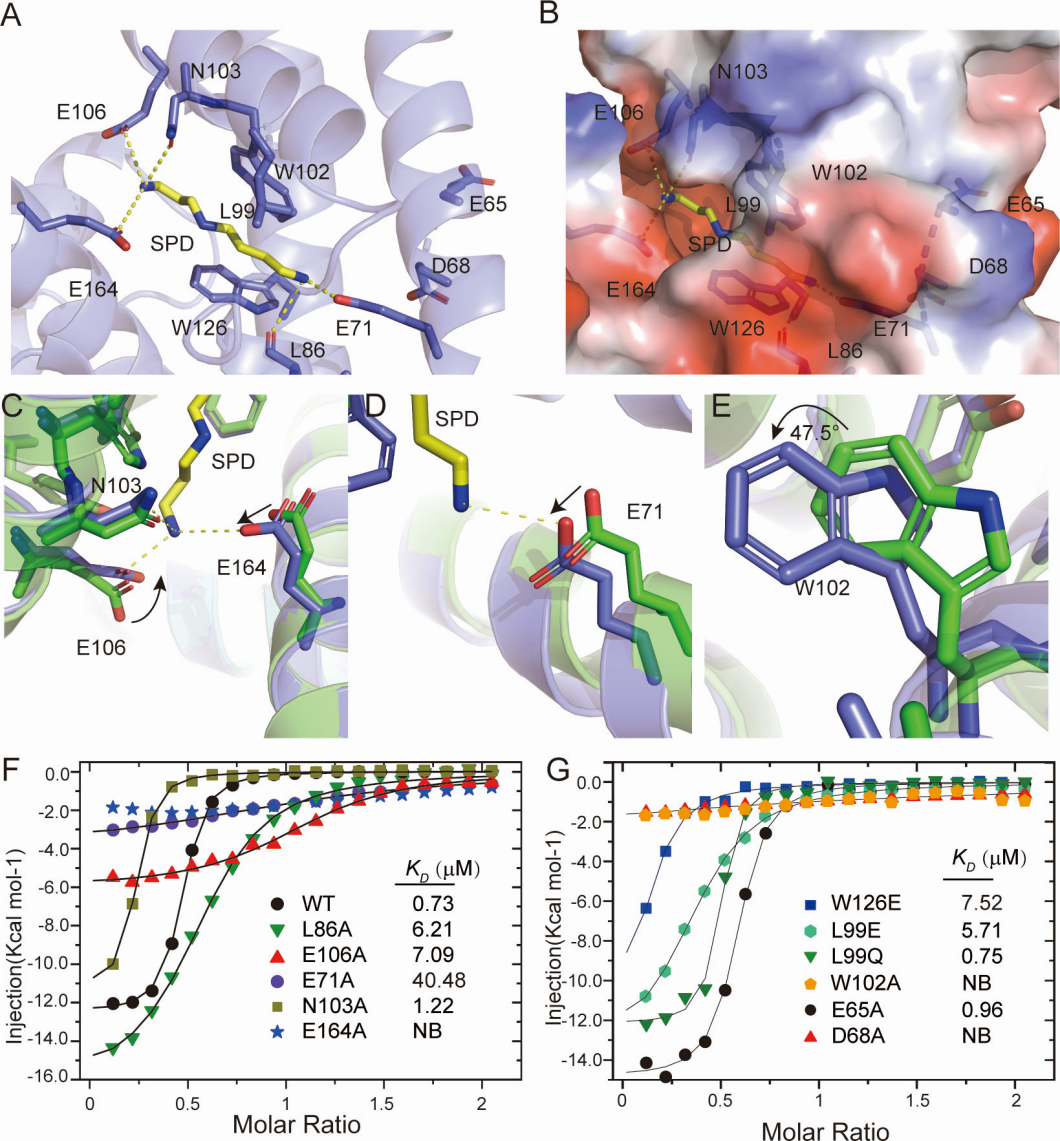
Interaction of spermidine with AmvR. (A, B) Detailed interactions between AmvR with spermidine. SPD-bound AmvR shown in light blue, with residues and spermidine are shown as the sticks. AmvR presents as a cartoon model (A) or electrostatic surface (B). (C–E) Superposition of the ligand binding pocket of *apo* and SPD bound AmvR. The side chain of E106 and E164 moved to form a hydrogen bond with the amino group of SPD (C), and α5 shifted to drive E71 to form a hydrogen bond with another amino group of SPD (D). The W102 rotated to form a hydrophobic interaction with spermidine (E). Residues in the *apo* or SPD-bound AmvR are depicted in green and light blue, whereas spermidine is represented in yellow. The hydrogen bonds are indicated by yellow dashes. (F) The hydrophilic residues identified in panel A were individually mutated to alanine, and their interactions with spermidine were assessed through ITC experiments. (G) The hydrophobic residues shown in panel A, along with hydrophilic residues forming the binding tunnel, were, respectively, mutated to alanine or hydrophilic amino acids, and their interactions with spermidine were also evaluated using ITC experiments.

The ligand-binding pocket consists of the residues in α4, α6–8, and loops between α5 and α6, which form a long narrow tunnel (Fig. 5A and B; Fig. S4A). The side chain of E164 and N103 in the α4 helix, along with the side chain of E106 in the α6 helix, moved to form hydrogen bonds with the amine group of spermidine (Fig. 5A). The polar residues collectively contribute to the recognition of the spermidine head, leading us to designate the pocket formed by E164 and E106 as an anchor site (Fig. S4B and C). Substitution at N103 slightly reduced the affinity, whereas mutation of the negative residue E106 led to a significant decrease in affinity. Additionally, AmvR^E164A^ eliminated spermidine binding, highlighting the importance of the anchor site (Fig. 5F).

Another amino group of spermidine formed a hydrogen bond with the side chain of E71, which underwent movement along the α6 helix and the carbonyl group on the main chain of L86 (Fig. 5D). Consequently, substitution of alanine at E71 disrupted this interaction with spermidine, leading to a significant decrease in affinity (Fig. 5F). The polar regions, comprising the main-chain carbonyl group and side-chain of E71, were identified as a recognition site in our study (Fig. S4B and C).

The hydrophobic interactions provided by W102, W126, and L99 are also crucial for spermidine binding, especially W102 at α6, which shifts and stabilizes spermidine (Fig. 5E). Substitution of W102 completely eliminated spermidine binding, emphasizing its crucial role in ligand recognition. In contrast, substitutions at residues W126 and L99 significantly decreased the affinity of spermidine for AmvR, further supporting the importance of W102 in maintaining ligand binding (Fig. 5G).

The negatively charged residues were distributed at both ends of the ligand-binding pocket, forming two negatively charged sites along with a narrow tunnel suitable for holding positively charged cell metabolites, such as polyamines (Fig. S4A through D). Mutations of all polar residues within the tunnel that interact with spermidine resulted in a reduction in the affinity of AmvR for spermidine (Fig. 5F). Negatively charged residues located at the terminus of the binding tunnel that do not directly interact with spermidine are crucial for spermidine binding. Mutation at D68 significantly reduced the affinity for spermidine, whereas mutation at E65, which was situated on the protein surface, did not have a similar effect (Fig. 5G). Additionally, substitutions such as L99 or W126 with negative residue like glutamate only resulted in a reduction, rather than elimination, in the affinity for spermidine by disrupting hydrophobic interactions (Fig. 5G). Notably, AmvR^L99Q^ demonstrated comparable affinity to the wild-type protein, likely due to its longer side chain of glutamine, which may help stabilize binding through alternative interactions, such as hydrogen bonds, which partially compensate for the hydrophobic interaction of leucine (Fig. 5G). These experimental findings underscore the importance of a negative environment in the binding tunnel for the recognition of polyamines, particularly spermidine. We hypothesized that the polar polyamines enter from one end of the narrow tunnel and finally reach the anchor site.

### Recognition of polyamine by AmvR

**Fig 6:**
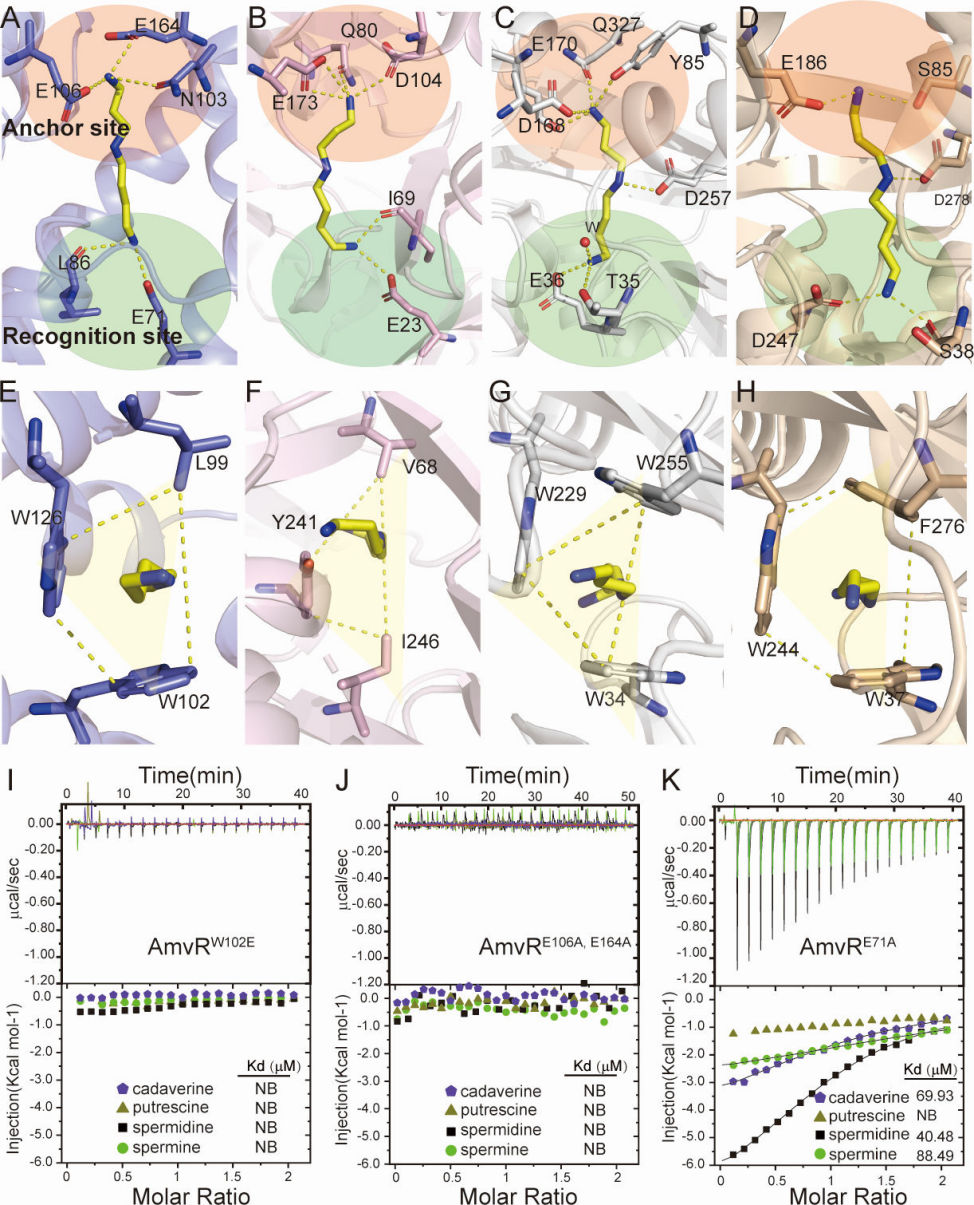
The recognition of spermidine in polyamine-binding proteins. The architecture of the recognition mode of spermidine in AmvR (A, E) was consistent with spermidine synthase (B, F; human spermidine synthase shown in pink, PDB: 2o07), spermidine transport (C, G; *E. coli* polyamine transport PotD shown in gray, PDB: 1POT) and putrescine receptor (D, H; *E. coli* putrescine receptor PotF shown in orange, PDB: 6YE8). The interacting residues are depicted in the sticks of the same color as mentioned earlier, and spermidine is represented by yellow sticks. (I-K) The results for different polyamines binding with mutants of AmvR containing AmvR^W102E^ (I), AmvR^E106A, E164A^ (J), and AmvR^E71A^ (K) from ITC experiments.

This recognition pattern, which utilizes the anchor site and recognition site to interact with spermidine, has also been observed in other polyamine-binding proteins, including polyamine transporter and spermidine synthase (Fig. 6). Recognition of polyamine amino groups by negatively charged residues has been observed in several proteins. In AmvR, E164 and E106 interact with one amino group (Fig. 6A), whereas in spermidine synthases, E173 and D104 perform similar roles (Fig. 6B) (31). In the polyamine transport protein PotD, D168 and E170 recognize the amino group (32), whereas in the putrescine receptor PotF, E186 is involved (Fig. 6C and D) (33). Additionally, glutamate residues or carbonyl groups from the main chain contribute to the recognition of other amino groups. For AmvR, E71 and the carbonyl group are involved (Fig. 6A), whereas for spermidine synthases, E23 and the carbonyl group are involved (Fig. 6A) (31). In PotD, E36 and water are responsible (32), whereas in PotF, D247 and the carbonyl group contribute to polyamine recognition (Fig. 6C and D) (33). These interactions are shown in the structural comparison presented in Fig. 6.

ITC demonstrated that the double mutation at E106 and E164, which constitute the anchor site, not only abolished binding with spermidine but also rendered AmvR completely unresponsive to other tested polyamine molecules (Fig. 6J). Unlike the anchor site, the E71A mutation at the recognition site exhibited differential effects on the affinity for the four tested polyamines. Specifically, the binding affinity for spermidine and spermine decreased approximately 50-fold, from 0.73 µM and 2.16 μM to 40.48 µM and 88.49 µM, respectively. In contrast, the affinity for cadaverine showed a more modest reduction of approximately 10-fold, decreasing from 5.88 μM to 69.9 µM (Fig. 6K).

We propose that E164 and E106 of AmvR form an anchor site critical for the binding of all polyamines, whereas E71 within the binding pocket acts as a recognition site to differentiate between various polyamines. The spatial arrangement between the negatively charged residues at the recognition site (E71) and the anchor site (E164 and E106) plays a pivotal role in determining the optimal polyamine length. In AmvR, this spacing is precisely tailored to accommodate spermidine, thereby facilitating its preferential binding. Consequently, the E71A mutation exerted a more pronounced effect on substrates with medium to long chain lengths, such as spermidine and spermine (Fig. 6K). Moreover, other polyamine-binding proteins such as PotD and PotF can form an additional hydrogen bond with the imino group, facilitating the recognition of diamines, a feature absent in AmvR (Fig. 6C and D). This structural distinction underlies the lower affinity of AmvR for putrescine and indicates that its binding specificity is finely tuned to respond preferentially to changes in spermidine concentration.

The hydrophobic interactions in the binding tunnel comprising W126, W102, and L99 robustly engaged the aliphatic chain of spermidine from various angles. This recognition and binding mode, corroborated by similar observations in other polyamine-binding proteins, underscores its conserved characteristics (Fig. 6E through H). Mutations at W102 to alanine or glutamate eliminated the binding affinity of all tested polyamines, illustrating the pivotal role of hydrophobic interactions in polyamine recognition (Fig. 6I). Mutations at either L99 or W126 to glutamate also markedly reduced the binding affinity of spermidine for AmvR, highlighting the importance of hydrophobic interactions in recognizing positive polyamines (Fig. 5G). In conclusion, the anchor site along W102 plays a pivotal role in polyamine binding to AmvR, with E71 distinctly augmenting its affinity for spermidine, thus establishing it as the preferred substrate.

## DISCUSSION

In this study, we delineated the molecular interactions between AmvR and its physiological substrate spermidine, providing insights into the molecular mechanism of substrate recognition. Despite their low molecular weight, polyamines are abundant amino acid-derived metabolites with short linear or branched structures (12). Polyamines play a pivotal role in bacterial pathogenesis (15). These small molecule metabolites are highly conserved and prevalent within cells (12). They affect bacterial function through their general biochemical properties or act as signaling molecules that engage with specific receptors at low concentrations. Elevated levels of polyamines, whether endogenously produced or externally acquired, can be detrimental (10, 12). Various efflux systems, including the small multidrug resistance (SMR) family, assist in polyamine transport (24). Our findings provide a basis for further investigations of cellular responses to changes in polyamine concentrations.

The TetR family regulators generally undergo marked conformational changes upon substrate binding (25). Size exclusion chromatography (SEC) revealed that the retention time of the AmvR-spermidine complex is substantially longer than that of *apo* AmvR, and this discrepancy became more pronounced as spermidine concentration increased (Fig. 4C). SEC results confirmed a significant conformational transformation of the dimeric form of AmvR upon spermidine interaction. Additionally, static light scattering revealed a reduction in radius after incubation with spermidine (Fig. S3A). Simultaneously, no significant changes were detected in the secondary structure based on circular dichroism spectrum analysis (Fig. S3C). However, crystal structure analysis indicated that the conformation of AmvR, when bound to spermidine, exhibited minimal variation from its *apo* state. Notably, the observed conformations may have been influenced by crystal packing constraints, potentially limiting the detection of conformational changes. Based on the results of the SEC experiments, we assumed that spermidine may affect the state of the AmvR dimer, which may have different conformations in solution (34). In response to the elevated spermidine concentrations, the DBD of AmvR may transit from a more compact form I to a relaxed form II, which we captured in crystal structures, leading to dissociation from the promoter region (34).

Structural conformational changes predominantly occur within the substrate-binding pocket, where the interaction with spermidine induces a reorganization of amino acids in α4 and α6, associated with α7–8 and the loop between α5 and α6. This reorganization forms a narrow tunnel that effectively accommodates positively charged polyamines through extensive hydrogen bonds, ensuring the specific recognition of polyamine molecules by AmvR, similar to other polyamine-binding proteins. The binding tunnel, delineated by W126, W102, and L99, leverages hydrophobic interactions to stabilize the polyamines. Unlike polyamine transporters, the substrate-binding pocket of AmvR does not form hydrogen bonds with the intermediate imino group of spermidines, highlighting its distinctive binding module (16). The polyamine pockets of 1POT and 6YE8 exhibited a hydrogen bond between the main carbonyl group and the intermediate imino group, facilitating the recognition of their native substrate and shorter putrescine (32, 33). This characteristic is absent in the AmvR proteins, which may account for the reduced binding affinity of AmvR for shorter polyamine molecules. Furthermore, the repositioning of E71 to α4 promoted the formation of a hydrogen bond, enhancing the recognition of spermidine and concurrently constraining the length of the substrate accommodated within the head recognition pocket.

Our experimental evidence supports a model in which AmvR modulates the efflux of polyamine (Fig. S5). At low intracellular concentrations of polyamines, AmvR occupies the intergenic region between *amvR* and *amvA*, thereby inhibiting AmvA efflux pump expression. In contrast, at elevated polyamine levels, these molecules interact with AmvR, triggering its dissociation from DNA and consequently promoting the expression of target genes. Increased transcription of *amvA* contributes to enhancing the polyamine efflux and safeguarding cellular integrity.

In summary, we elucidated the mechanism by which AmvR recognizes polyamines and demonstrated that its DNA-binding activity is influenced by spermidine to modulate AmvA expression. Unveiling the crystal structure of AmvR represents an initial step toward understanding the structural basis underlying the function of regulatory proteins that govern drug resistance and virulence in pathogens.

## MATERIALS AND METHODS

### Gene clone and protein expression

The gene of AmvR (a.a. 4–192; NCBI reference sequence: WP_000323807.1) was amplified from the *A. baumannii* genome using PCR and subsequently cloned into a modified pET-28a (+) expression vector (Novagen) with an N-terminal 6* His tag followed by maltose-binding protein (MBP) and a non-canonical TEV protease cleavage site (E-N-L-Y-F-Q-Cys) (35). All mutations used in this study were introduced using standard PCR procedures, digested by Dpn I, and verified by DNA sequencing.

The verified plasmid was expressed in *E. coli* strain BL21 (DE3) using the heat shock method. Cells were grown at 37°C in Luria Bertani (LB) medium supplemented with kanamycin. Protein was induced with 0.2 mM isopropyl β-D-thiogalactoside (IPTG) when the OD_600_ was 0.7, and the cells were harvested after overnight incubation at 16°C and 180 rpm. The collected cell pellet was resuspended in lysis buffer A (500 mM NaCl, 20 mM Tris-HCl, pH 8.0). The resuspended cells were lysed by sonication followed by centrifugation at 13,000 rpm for 1 h. The supernatant containing soluble AmvR protein was incubated with Ni-Sepharose affinity beads (GE Healthcare) and washed with wash buffer B (20 mM imidazole, 500 mM NaCl, 20 mM Tris-HCl, pH 8.0). Recombinant AmvR protein was eluted with elute buffer C (200 mM imidazole, 500 mM NaCl, 20 mM Tris-HCl, pH 8.0). The His-MBP tag was then removed by TEV protease, and the protein was further purified using a HiLoad 16/600 Superdex 200 pg column (GE Healthcare) with buffer D (200 mM NaCl, 20 mM Tris-HCl, pH 8.0). Purified protein samples were rapidly frozen in liquid nitrogen and stored at −80°C for future use.

### Crystallization

The protein samples used for crystallization were mixed with precipitant in a 1:1 ratio at concentrations of 5 mg/mL and 10 mg/mL. For the ligand-bound crystal, the AmvR was incubated with 5 mM spermidine for 30 min at room temperature before crystallization. The crystallization experiments were conducted using the sitting drop method at 16°C. All crystals were immersed in cryoprotectants derived from the mother liquors supplemented with 15%–30% glycerol before rapid freezing in liquid nitrogen.

### Data collection and structure determination

The diffraction data for all crystals were collected at a wavelength of 0.9791 Å at beamline 19U (BL19U1) and 02U (BL02U1) at Shanghai Synchrotron Radiation Facility (SSRF), China (36). Following processing with AutoPX (37), the *apo* AmvR structure was solved through molecular replacement in PHASER (38) using the AlphaFold model as the search model and subsequently manually refined and built using Coot (39). The AmvR-SPD structure was determined via molecular replacement in PHASER with our structure of *apo* AmvR as the search model. All structure was refined by PHENIX (40). Table 1 summarizes the statistics for data collection and structure refinement. All Figures were prepared using PyMOL (The Pymol Molecular Graphics System, Schrödinger).

### Isothermal titration calorimetry

The AmvR^WT^ or mutant proteins were purified with affinity chromatography followed by size-exclusion chromatography and buffer-exchange with buffer D. The polyamines were also dissolved in Buffer D. The ITC experiments were performed at 20°C using a MicroCal PEAQ-ITC (Malvern Panalytical Ltd) using 20 injections of 2 µL or 25 injections of 1.6 µL. A solution of 100 µM AmvR wild-type or mutants was loaded into the cell, whereas a 1 mM solution of polyamines was loaded into the syringe. The measured heat changes of the binding reactions were integrated and analyzed using the standard “one set of sites” model implemented in the Origin software package (OriginLab) to determine the binding stoichiometry (N-value) and the equilibrium dissociation constant (Kd).

### Electrophoretic mobility shift assay

The DNA used for EMSA was amplified from PCR with FAM-labeled prime 1 and prime 2 synthesized by General Biol. Specific DNA was amplified from PCR with prime 3 and prime 2. The purified wild-type and mutated proteins were incubated with 0.2 µM FAM-labeled DNA 1 in a binding buffer E (100 mM HEPES-Na, pH 8.0, 100 mM KCl, 50 mM MgCl_2_, 5% glycerol, 10 mM DTT, 10 mM EDTA), and 0.1 mg/mL bovine serum albumin (BSA) at various concentrations. After 30 min of incubation at room temperature, the samples were loaded onto the 6% native Tris-acetate EDTA (TAE)-acrylamide gels. Electrophoresis was then conducted in TAE buffer at 140 V for 60 min, followed by imaging analysis using Typhoon FLA 9500 (GE Healthcare).

### Size-exclusion chromatography assays

The purified AmvR^WT^ or AmvR^G162R^ were buffer exchanged to buffer F (200 mM NaCl, 20 mM Tris, pH 8.0, 5 mM DTT) before being applied into a Superdex 75 Increase 10/300 Gl column (GE Healthcare) pre-equilibrated with buffer F. The collected fractions were analyzed using SDS-PAGE. To validate the conformational change after binding with spermidine, AmvR^WT^ was incubated with two and ten times the concentration of spermidine, respectively. The samples were then incubated in buffer F on ice for 1 h, with a total volume of 200 µL, respectively, and applied to a Superdex 75 Increase 10/300 Gl column (GE Healthcare) pre-equilibrated with buffer F. The gel filtration standard components (thyroglobulin, 670 kDa; γ-globulin, 158 kDa; ovalbumin, 44 kDa; myoglobin 17 kDa; vitamin B12, 1.35 kDa) were applied to a Superdex 75 Increase 10/300 Gl column (GE Healthcare) pre-equilibrated with buffer F as a control.

### DNase I footprinting assay

The 5′-FAM-labeled target DNA 1 was PCR amplified using 5′-FAM-labeled primers. In a 200 µL reaction system, 2000 ng of labeled DNA fragment and 18 µM AmvR (final concentration) were mixed (in the control experiment, bovine serum albumin was used instead of AmvR) in 10 mM HEPSE-Na (pH 8.0) with 10 mM MgCl_2_, 1 mM CaCl_2_, 0.4 mM dithiothreitol, 100 mM KCl, and 5% glycerol, and incubated at room temperature. After 30 min, 0.15 U or 0.3 U of RNase-free DNase I was added for digestion at 25°C for 1 min. The reaction termination, precipitation, and analysis procedures were done as reported (41).

### Fluorescence reporting assay

The promoter regions of *amvR* and *amvA* were amplified from the genome and cloned upstream of EGFP in the modified pUC19 plasmid. These resulting constructs were designated as pUC19-P*amvR*-EGFP and pUC19-P*amvA*-EGFP, respectively. Additionally, based on these original constructs, the J23119 promoter followed by the sequence of *amvR* was inserted upstream of the promoter region. These plasmids were named pUC19-*amvR*-P*amvR*-EGFP and pUC19-*amvR*-P*amvA*-EGFP. The plasmids were individually transformed into *Escherichia coli* DH5α, and the bacteria harboring the reporter plasmids were cultured to the early post-exponential phase in LB medium supplemented with ampicillin at 37°C for 12 h until reaching an OD_600_ of 0.6. Subsequently, the cultures were transferred to 96-well plates and diluted with deionized water or PBS. Fluorescence signals (excitation at 485 nm/emission at 535 nm) and OD_600_ were measured using a SpectraMax iD3. The fluorescence units (RFU) were normalized, and the background-corrected reporter fluorescence values were calculated by subtracting the RFU/OD_600_ (fluorescence = fluorescence units/OD_600_). The data reported are the averages and standard deviations (SD) from at least three independent experiments.

## Data Availability

Coordinates of the X-ray crystal structures have been deposited in the RCSB PDB (https://www.rcsb.org) with the following accession numbers: *apo*-AmvR (8WP6) and AmvR-SPD (9IPT). Other data are available upon reasonable request.

## SUPPLEMENTAL MATERIAL

Supplemental table and figures mbio.00081-25-s0001.pdf Table S1 and Figures S1-S5.

Raw data of EMSA mbio.00081-25-s0002.pdf Raw data of EMSA, related to Fig. 2A and B.

ASM does not own the copyrights to Supplemental Material that may be linked to, or accessed through, an article. The authors have granted ASM a non-exclusive, world-wide license to publish the Supplemental Material files. Please contact the corresponding author directly for reuse.
