# Supplementary material for: Structural basis for spermidine recognition and modulation of Acinetobacter baumannii multidrug efflux regulator AmvR
Source: mBio. 2025 Mar 31;16(5):e00081-25. doi: 10.1128/mbio.00081-25 (PMC12077194; doi:10.1128/mbio.00081-25)
Supplement: Supplemental table and figures — Table S1 and Figures S1-S5. [file mbio.00081-25-s0001.pdf]

Supplementary Figures for

Structural Basis for Spermidine Recognition and Modulation of *Acinetobacter baumannii* Multidrug Efflux Regulator AmvR

Na Wang[a], Xu Wang[a], Mengxiang Zhou[a], Qingsong Lu[a], Yaling Xu[a], Ying Wang[a], Haiyun Wang[a], Beibei Yang[a], Shibing He[a], Liuliu Xu[a], Jie Li[a], Honghua Ge[a#], Jinming Ma[a#]

Institute of Health Sciences and Technology, Institutes of Physical and Information Technology, Anhui University, Hefei 230601, China

# Correspondence email: hhge@ahu.edu.cn, jmma@ahu.edu.cn

Supplementary Table 1 DNA sequence and primers used in EMSA

ID	Sequence
Fam_DNA 1	Fam-<br>aatcatttctctgcgttgatcgcgattaagataggccatgtttttttacctgactgaattcgacacaaattacattaaaa<br>ctaaaagttggacaagtgtccagttgtgtatataataaccatgatttgaacacatgtccaagttttggatattgaaga<br>gcgttatgcaaaaaaaatggttaatcctgacaattatcgtcctcatttatttaccagttacgattgatgcaacggtaat<br>gcatgttgcaacaccatctttaagtgcagcattgaatttaactgccaatcagcttttatggattattgatatttattcact<br>gattatggcgggtttgattttgccgatgggtgcac
DNA 1	aatcatttctctgcgttgatcgcgattaagataggccatgtttttttacctgactgaattcgacacaaattacattaaaa<br>ctaaaagttggacaagtgtccagttgtgtatataataaccatgatttgaacacatgtccaagttttggatattgaaga<br>gcgttatgcaaaaaaaatggttaatcctgacaattatcgtcctcatttatttaccagttacgattgatgcaacggtaat<br>gcatgttgcaacaccatctttaagtgcagcattgaatttaactgccaatcagcttttatggattattgatatttattcact<br>gattatggcgggtttgattttgccgatgggtgcac
Prime 1	Fam-aatcatttctctgcgttgatcgcg
Prime 2	gtgcacccatcggcaaaatcaaacccg
Prime 3	aatcatttctctgcgttgatcgcg

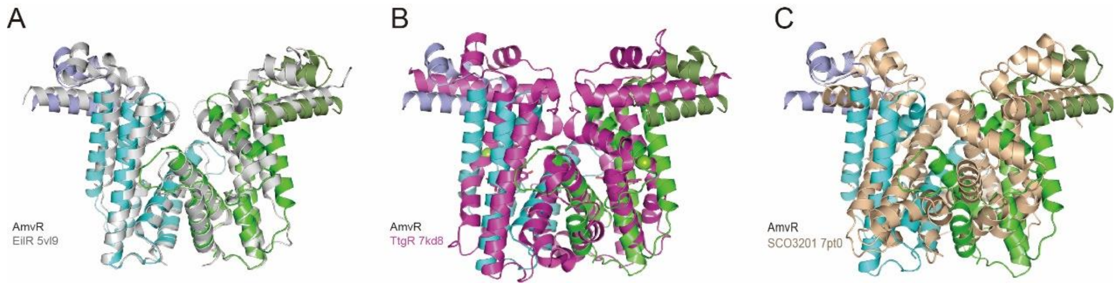

Supplementary Figure 1. AmvR was a TetR family regulator.

A-C) Structure alignment of *apo* AmvR with EilR (A), TtgR (B) and SCO3201 (C). AmvR is represented in the same color as in Figure 1, while EilR is shown in grey, TtgR in pink, and SCO3201 in dark orange.

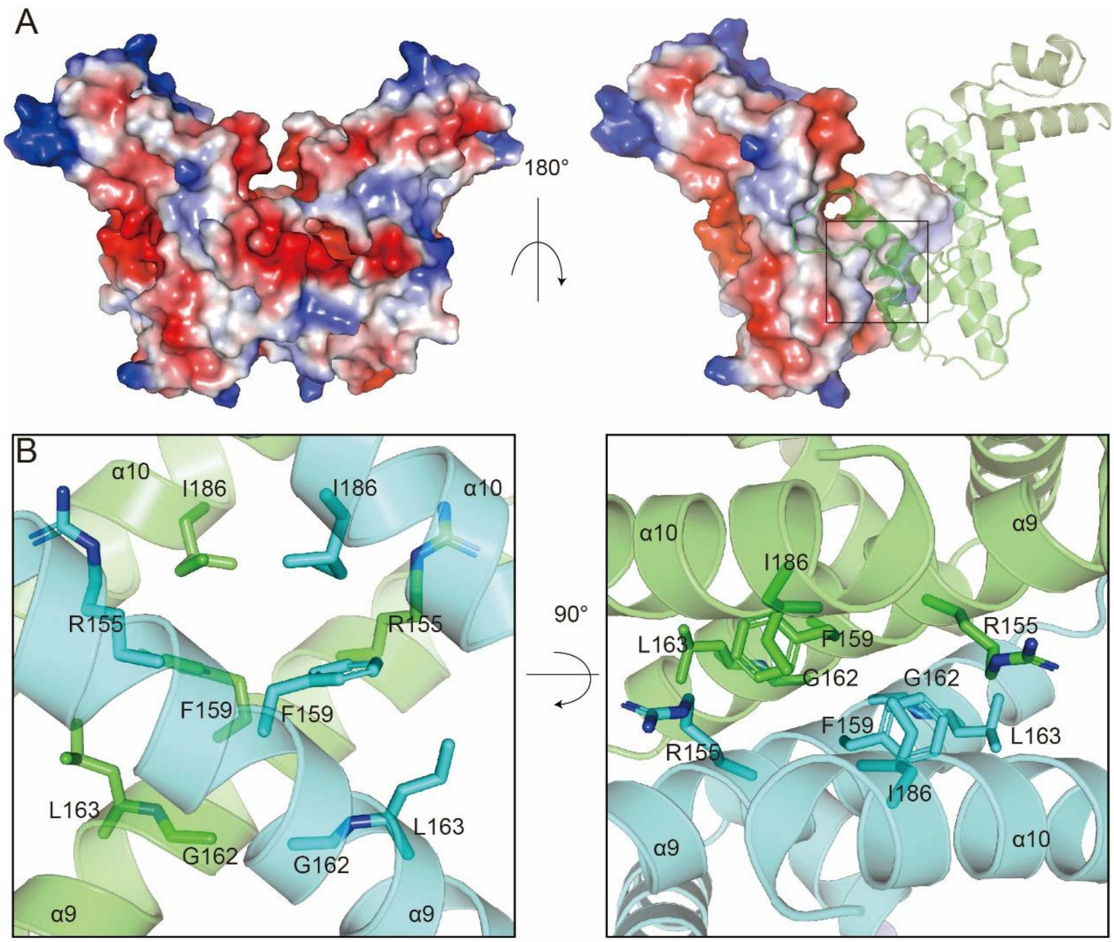

Supplementary Figure 2. The dimer interface of *apo* AmvR.

A) Two surface view 180° apart of *apo* AmvR color-coded with the electrostatic potential (red, negatively charged; blue, positively charged), with one protomer show in cartoon to reveal the interface of dimer(right). The color shown as Figure 1.

B) Zoom-in views, 90° apart, showing the hydrophobic interaction of the dimer, with residues depicted in stick representation.

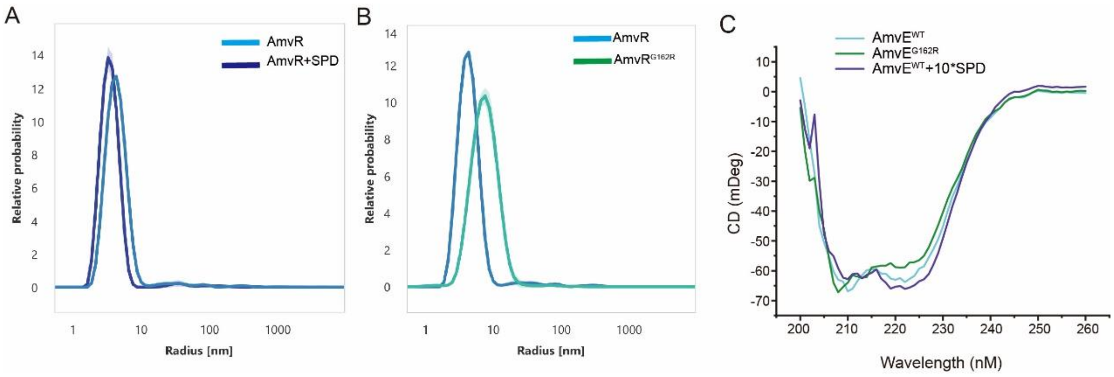

Supplementary Figure 3. Illustrates the conformational change of AmvR induced by the binding of SPD.

A) Static light scattering analysis of *apo* AmvR (shown in cyan) and SPD bound AmvR (shown in indigo), conducted using Prometheus Panta instrument (NanoTemper). **B**) Static light scattering analysis of AmvR^{WT} (shown in cyan) and AmvR^{G162R} (shown in green), conducted using Prometheus Panta instrument (NanoTemper). The x-axis displays the logarithm of molecular diameter (Log [D (nm)]), while the y-axis represents the normalized scattering intensity.

C) Circular dichroism spectrum analysis of *apo* AmvR^{WT} (shown in cyan), SPD-bound AmvR (shown in indigo) and AmvR^{G162R} (shown in green), conducted using spectropolarimeter (BioLogic, MOS-500).

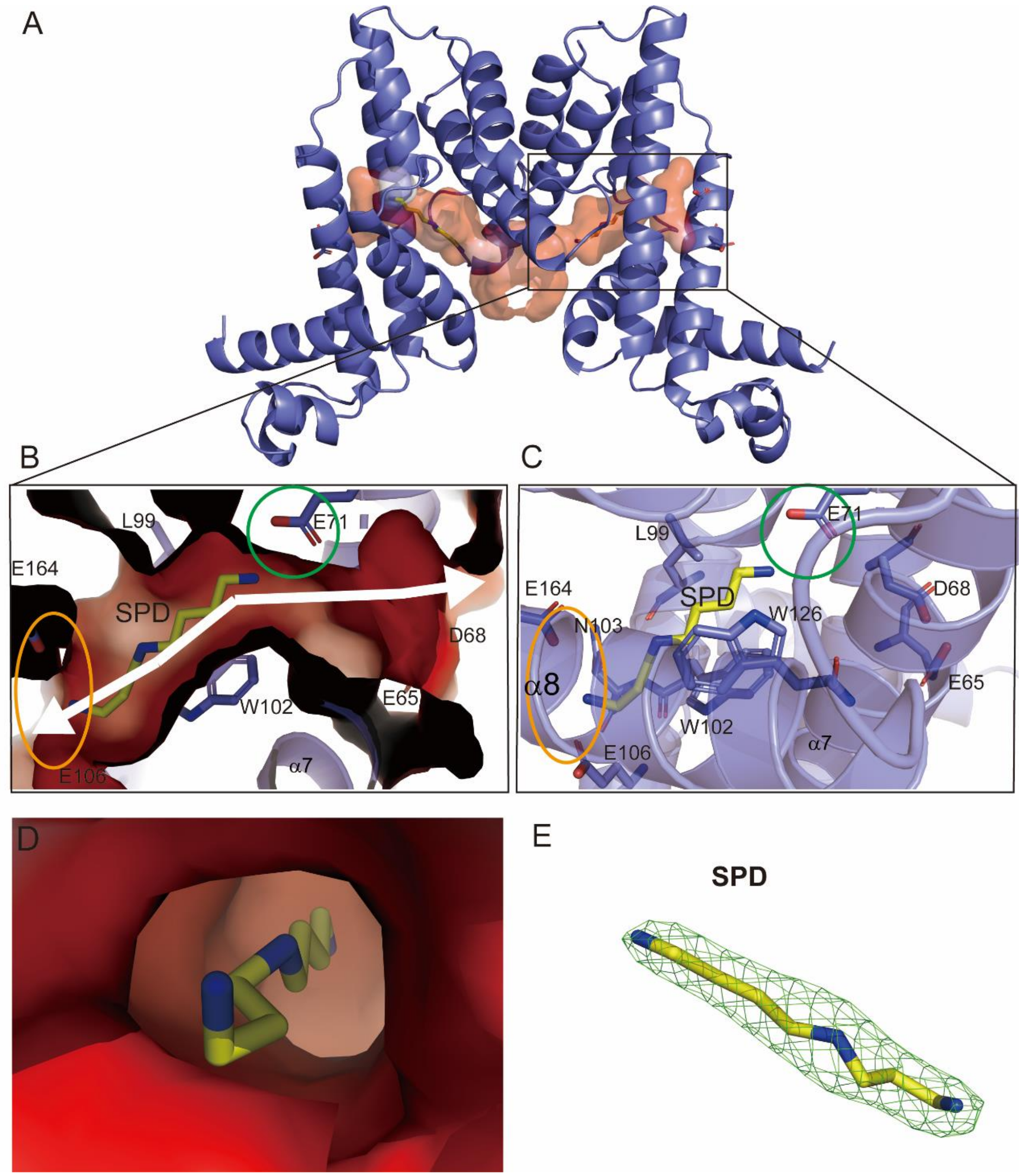

Supplementary Figure 4. Spermidine lay on the narrow channel of AmvR.

A) The electrostatic surface of SPD-bound AmvR, spermidine was laid on the negative narrow channel of LBD of AmvR.

B) A cross-section of the narrow binding tunnel for spermidine. SPD was shown as yellow sticks, with the surrounding residues represented as light blue sticks.

C) The substrate binding pocket of AmvR is shown in cartoon mode from the same view as (B), with residues depicted in light blue sticks. Anchor site shown in orange circle and recognition site shown in green circle.

D) Zoom-in view of spermidine binding pocket on one side of the binding tunnel;

E) Chemical nature and density map of spermidine. The $2|Fo|-|Fc|\sigma$-weighted map is contoured at 1.5σ.

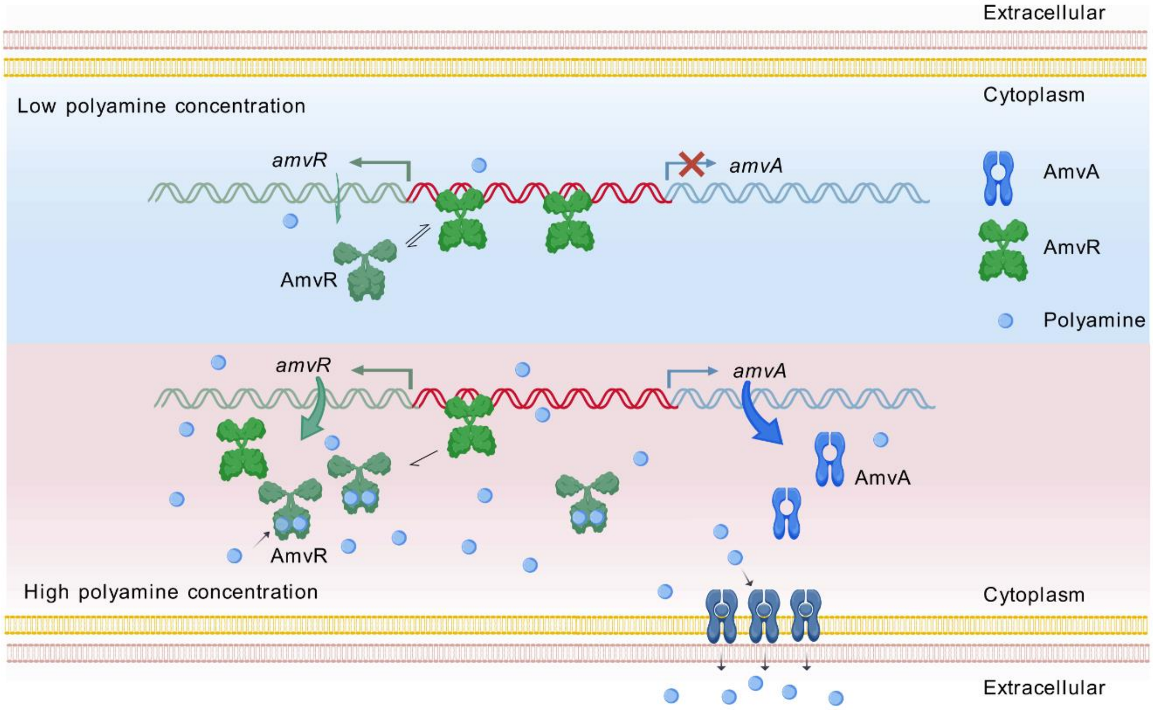

Supplementary Figure 5. Proposed model for AmvR-mediated regulation of AmvA in *A. baumannii*. The blue background represents conditions of low intracellular polyamine concentrations, while the pink background represents high intracellular polyamine concentrations. Under low polyamine levels, AmvR (green proteins) binds to the intergenic region (red) between *amvR* and *amvA*, repressing the transcription of both genes (indicated by crosses). As polyamine levels (blue circles) increase, polyamines bind to AmvR, inducing a conformational change that causes it to dissociate from the DNA, thereby permitting the transcription of both *amvA* and *amvR* (indicated by arrows). The AmvA efflux pump (blue proteins) then exports polyamines out of the cell, reducing intracellular polyamine levels. The figure was created with BioGDP.com(1)

Reference

1. Jiang S, Li H, Zhang L, Mu W, Zhang Y, Chen T, Wu J, Tang H, Zheng S, Liu Y, Wu Y, Luo X, Xie Y, Ren J. 2025. Generic Diagramming Platform (GDP): a comprehensive database of high-quality biomedical graphics. Nucleic Acids Res 53:D1670-d1676.
