# Supplementary figures and images for: Structural basis for spermidine recognition and modulation of Acinetobacter baumannii multidrug efflux regulator AmvR
Source: mBio. 2025 Mar 31;16(5):e00081-25. doi: 10.1128/mbio.00081-25 (PMC12077194; doi:10.1128/mbio.00081-25)

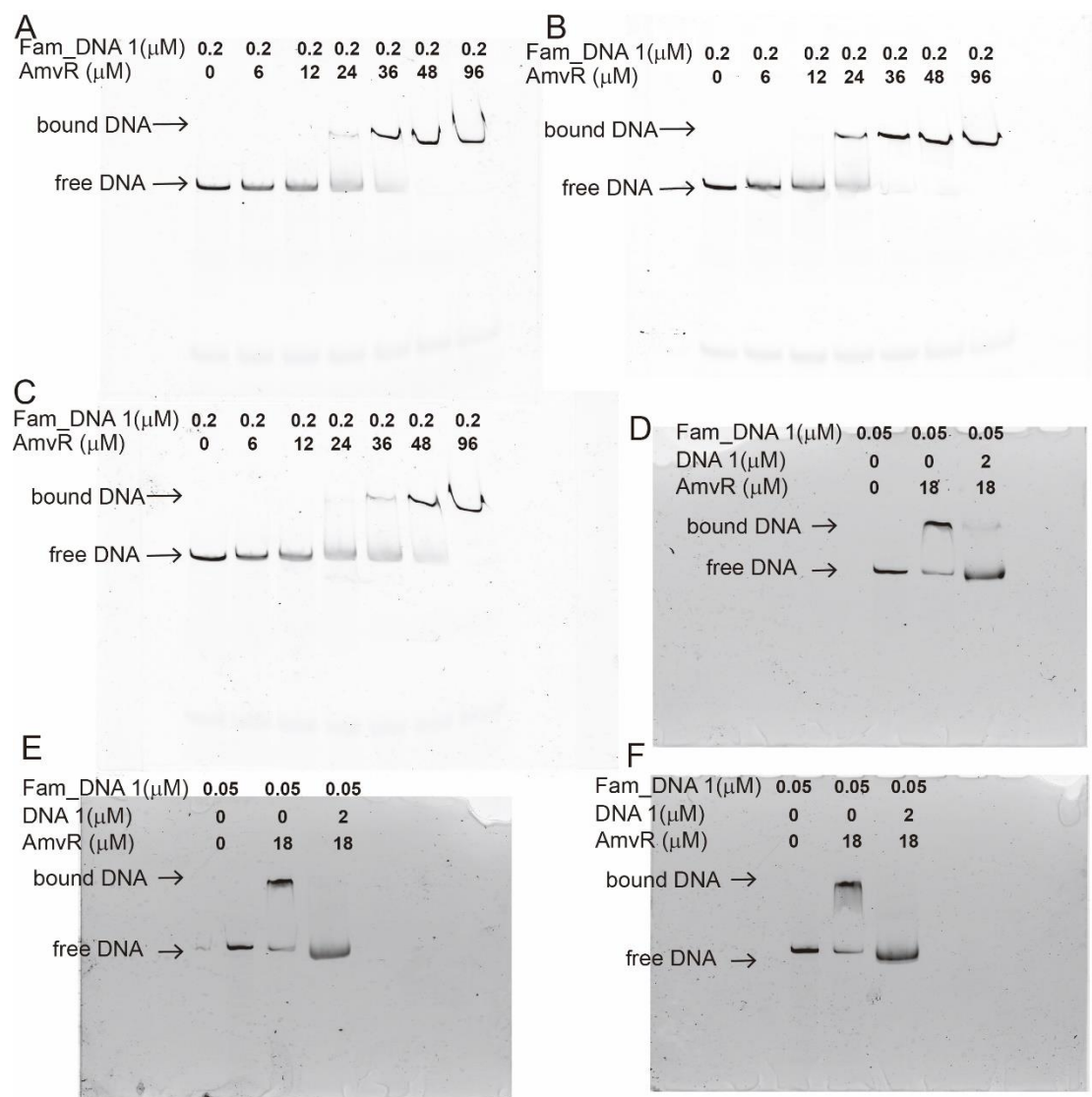

**Related to Figure 2A and B: Uncropped PAGE gels of EMSA.**

Supplement: Raw data of EMSA — , related to Fig. 2A and B. [file mbio.00081-25-s0002.pdf]
